# Food choices for weight loss: what dietary strategies would people use?

**DOI:** 10.1017/S0007114523002726

**Published:** 2024-04-14

**Authors:** Luana Giacone, Michael Siegrist, Christina Hartmann

**Affiliations:** ETH Zurich, Department of Health Sciences and Technology, Consumer Behavior, Universitaetstrasse 22, CH-8092 Zurich, Switzerland

**Keywords:** Weight loss strategies, Nutrition, Food choice, Dietary strategies

## Abstract

Previous observational research showed that one of the most common strategies used to lose weight is to avoid or restrict the consumption of specific food items. However, the question of how people behave and implement strategies in actual decision-making situations involving food choices for weight loss purposes remains inconclusive. This experimental study using a food buffet aimed to examine people’s different dietary strategies and motives for selecting foods for an entire day for weight loss purposes compared with a normal-day (ND) food selection. A total of 111 participants (55 % women) had to choose foods for both a ND and a weight loss day (WLD) (within-study design). Kilocalories and nutrients were calculated based on the weights of the foods selected, and food choice motives were assessed using a questionnaire. The results showed that for weight loss purposes, the participants selected more vegetables (both sexes) and unsweetened beverages (only men) while reducing their choices of high-fat and high-energy products (both sexes). Participants’ food choices in both conditions (ND and WLD) differed from the official nutrition recommendations. They chose less carbohydrates and fibres and more fat and sugar than recommended. Health, kilocalories and nutrient content (carbohydrates, sugar, fat and protein) were more important food choice motives for weight loss purposes than for a ND food selection, while taste became less important. In conclusion, the participants appeared to be well capable of implementing several appropriate dietary strategies. Further research is needed to explore strategies to help them maintain these dietary changes over the long term.

For many years now, people’s desire to control their body weights seems to steadily increase, even in non-clinical settings and situations^(1,2)^. More than 40 % of adults worldwide reported some diet attempts in their lives^(3)^. Nevertheless, a high percentage of the global population is still overweight or obese because of an imbalance between energy intake and energy expenditure^(4)^, despite people’s increased desire to control their body weights. Therefore, a better understanding of people’s current dietary weight loss strategies may facilitate more effective weight management practices.

Weight loss can be achieved through different strategies. The most common weight loss strategies are changing one’s diet by restricting energy intake and increasing exercise^(5–8)^. Further dietary strategies include increasing the fibre content of one’s diet, omitting certain food categories (e.g. sweets and alcohol) or increasing one’s intake of certain food groups (e.g. fruits and vegetables) (for a review, see Ramage *et al.* ^(8)^). Changing the macronutrient composition of the diets (e.g. low-carbohydrate, high-protein or low-fat diets) is a further method used for weight management^(5,7,8)^. It is not possible to state with certainty how promising the various approaches are, but it is assumed that variations in weight loss with different macronutrient diets are probably attributed to differences in adherence^(5,9)^. Previous studies primarily examined weight loss strategies by using overweight or obese adults as the participants, particularly those participating in weight loss interventions, or analysed samples predominantly consisting of women^(8,10–13)^.

Observational studies^(2,14–16)^ showed that the general population avoided or restricted their consumption of specific foods when trying to control their weights. For example, they drank less alcohol and ate less fatty and sugary foods, less junk/fast food and high-carbohydrate foods, or less meat while eating more fruits and vegetables and consuming more low-energy foods and beverages (for a review, see Santos *et al.* ^(3)^). Further strategies involve skipping meals entirely and/or drinking a lot of water, while men are more likely to skip meals and less likely to drink more water compared with women^(2,15)^. Most of these observational studies of the general population revealed weight control strategies and practices which were collected mainly by using questionnaires and interviews^(2,3,14–16)^ rather than observing actual food choice decision-making situations. For instance, one study collected data utilising a 24-h dietary recall and examined whether different weight loss strategies were associated with the consumption of sugar-sweetened beverages (SSB) and snacks, as well as values related to food consumption^(17)^. Another study that tested multiple weight management strategies found that some strategies were more prevalent, while others were less common in the general population^(18)^. For example, the general population used the strategy of consuming low-energy beverages (water and unsweetened beverages) more often than substituting foods they craved with more nutritious ones (fruits or vegetables instead of sweets or salty snacks)^(18)^. All these previous studies lead to a better understanding of consumers’ eating behaviour if they desire weight loss. However, it is not well understood how consumers implement dietary strategies for weight loss in real-life food choice decisions and how these choices differ from those made without weight loss intention. Therefore, little is known about the actual use and implementation of different weight loss strategies in real-life food choice decision-making situations. Furthermore, it is important to identify potential dietary misconceptions about proper weight loss strategies. Consequently, the primary aim of the present study was to fill this knowledge gap by investigating in a more applied manner which dietary weight loss strategies are used by people when choosing meals for the entire day (weight loss day (WLD)) compared with a normal-day (ND) food selection.

## Food choice motives

Food choices are influenced by a diverse range of motives, and weight control can be one of them^(19,20)^. However, for the general population, taste is the most important food choice motive, followed by costs, nutritional values and convenience^(19)^. Previous research with overweight and obese individuals showed that the level of importance of food choice motives differed between weight-stable individuals and weight loss maintainers^(21)^. For weight loss maintainers, health and the belief that food was low in kilocalories (kcal) were more important food choice motives than for the weight-stable group^(21)^. As food choice motives shape which food decisions people make, a change in food choice motives should lead to a change in food decisions. Therefore, an understanding of the motives that may drive the general population’s food choices for weight loss purposes is critical for developing weight management strategies that promote long-term success. Following this, the second aim of this study was to examine whether the level of importance of different food choice motives would vary between a WLD and a ND without any weight loss intention.

## Methods

### Participants

The study participants were recruited through the Consumer Behavior Experimental Panel. The participants had to be at least 18 years of age and fluent in German. They should not suffer from any food allergies or intolerances, not follow a vegan diet, not have a nutritional background, and have the desire to lose weight or maintain it. Each participant was rewarded with 40 CHF for participating in this 40–60 min study. The required sample size for medium effects of 0·4 with a power of 0·8 consisted of fifty-four participants for each sex^(22)^. A total of 116 people took part in this study. The data from the participants who confused the two study conditions (*n* 3) or did not understand the instructions properly (*n* 2) were excluded from the analysis. Therefore, the data from 111 participants were analysed. Of these participants, 55 % were female, the mean age was 46 years (sd = 14, range = 19–70 years) and 65 % had a higher educational degree (Table 1).

**Table 1.** Descriptive characteristics of study participants (*n* 111), separated by sex and comparison to the general Swiss population

		Total (*n* 111)			Men (*n* 50)			Women (*n* 61)			Census 2022	
											Men	Women
	Range	%	M	sd	%	M	sd	%	M	sd	M or %	M or %
Age (years)	19–70		46	14		45	13		46	15	42	44
Education											13	
Low (%)		1			2			0				15
Middle (%)		34			36			33			40	43
High (%)		65			62			67			47	42
BMI (kg/m^2^)	16·54–36·29		24	4		25	3		24	4	26	24
Underweight (%)	< 18·5	3			2			3			1	5
Normal weight (%)	18·5–24·9	62			59			63			48	62
Overweight (%)	25–29·9	28			31			27			39	23
Obese (%)	≥ 30	7			8			7			12	10
Energy needs (kcal)	1780–3906		2530	487		2928	375		2205	283		
Dieting attempts (yes, %)		74			58			87				

Educational level was split into three categories: low = no education, primary and lower secondary school; middle = vocational school; high = higher secondary school, college and university.

Energy needs per day were calculated by multiplying the BMR by physical activity levels.

All participants were informed about the tasks and had to give their written consent before starting the experiment. This study was conducted according to the guidelines laid down in the Declaration of Helsinki, and all procedures involving human subjects were approved by the ethics committee of ETH Zurich (EK 2022-N-61). Written informed consent was obtained from all participants.

### Experimental procedure

The study was conducted between May and July 2022 in Zurich, Switzerland. The participants were individually invited to the study room, where they were introduced to the experimental food buffet. The buffet consisted of replica (fake food) and the packaging of real food items (e.g. yogurt cups or cereals). Fake foods are 3D models of real foods moulded from plastic which can be reused, avoid food waste and create a controlled buffet for every participant as the food always looks the same. Previous studies have proven the fake food method as valid and reliable for assessing food choices in a well-controlled environment^(23,24)^.

**Fig. 1.**
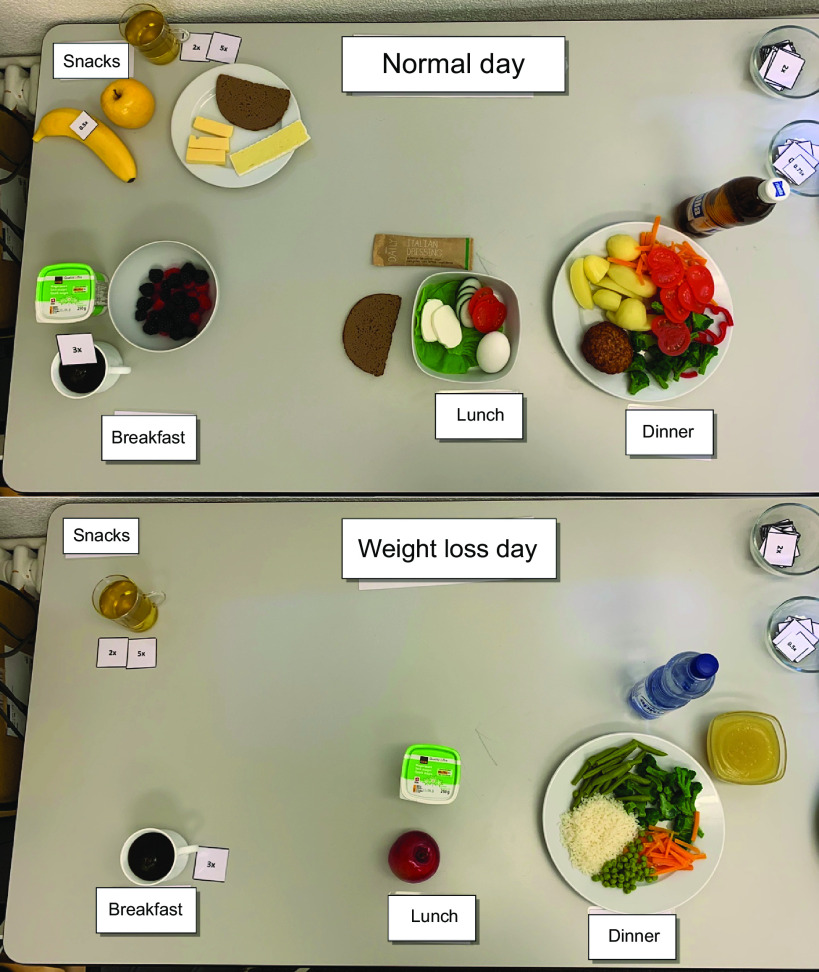
Food selection of a female participant, with 2004 kcal for the ‘normal day’ (top) and 1609 kcal for the ‘weight loss day’ (bottom).

The experiment included two conditions with a within-study design – a ‘normal day’ (ND) and a ‘weight loss day’ (WLD). Each participant completed both conditions in a quasi-randomised design. The participants were instructed to serve themselves breakfast, lunch, dinner and snacks as they would eat on a usual day (ND). In the other condition, they were instructed to serve themselves breakfast, lunch, dinner and snacks as they would eat if they had the wish to lose weight (WLD). Between the two conditions, the participants were asked to fill out the first part of a questionnaire on a tablet in a separate room. After the second condition, they were asked to finish the questionnaire. Meantime, the investigators took pictures of the assembled meals (see Fig. 1), weighed the continuous components (e.g. rice, pasta and vegetables) and counted the pieces of the single food items (e.g. bread, meat and sweets).

### Food buffet

**Fig. 2.**
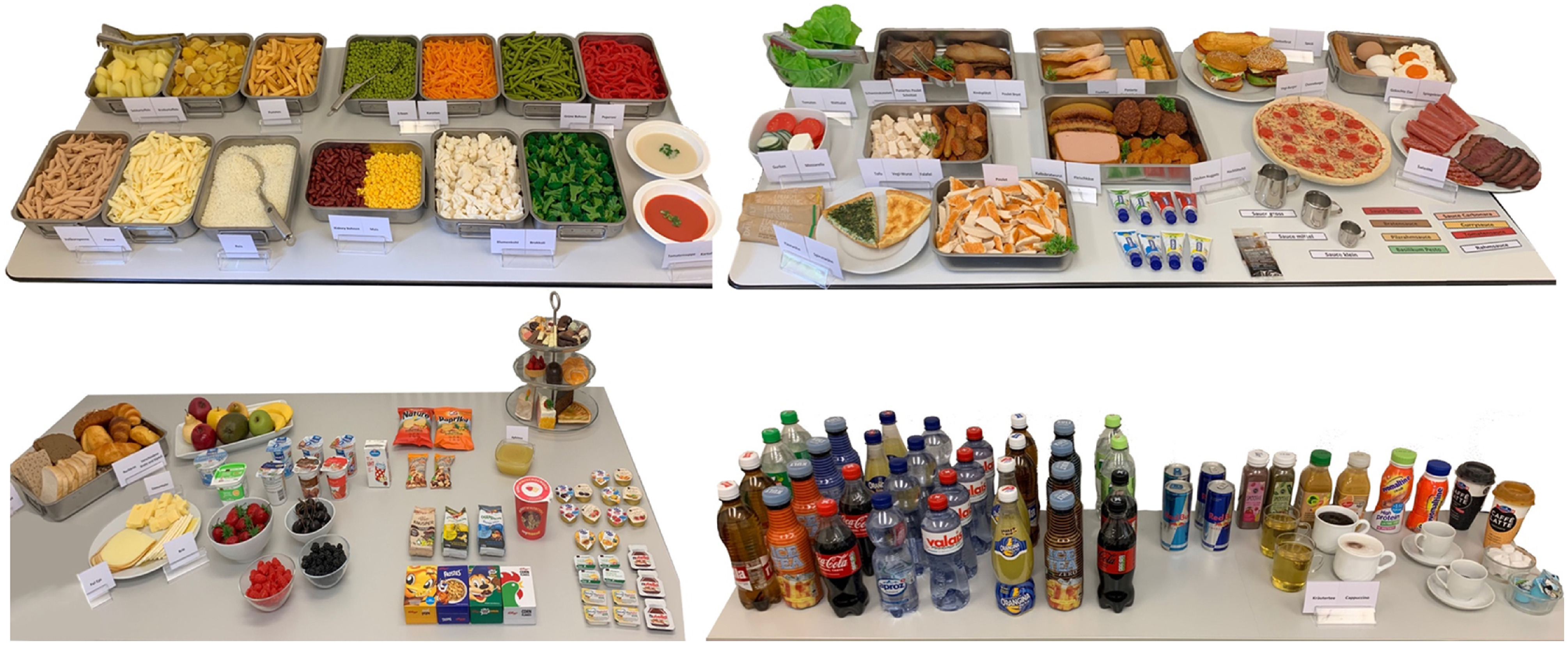
Food buffet containing ninety-two fake food items and sixty real food items.

The food buffet comprised 152 different food items (see Fig. 2). Of these, ninety-two items were fake foods^(23)^, and sixty items were packages of real foods, presented as single package portions (e.g. yogurt), which were purchased from local retailers. Except for alcoholic beverages, all food categories were represented (e.g. beverages, starchy foods, vegetables, fruits, meat and fish, oils and fats, sweets, and salty snacks). To estimate the total energy and macronutrients of the meals more precisely, sauces (e.g. tomato sauce and basil pesto) in three different sizes, salad dressings (French and Italian) and cold sauces (ketchup, mayonnaise, mustard and tartar) were also provided. Additionally, multiplication cards (e.g. 0·25×, 2×, 3·75×) were put at the participants’ disposal so that they could precisely choose the amount of a single food item (e.g. for tea, coffee or apples). The authenticity of the whole fake food was assessed, ranging from 1 = *not realistic at all* to 6 = *very realistic.* Overall, the food buffet was rated as authentic (M = 4·91, sd = 1·02).

The weights of the fake food items were converted by a conversion factor to obtain the real weights of the foods. For further information about the conversion factors, see previous publications^(23,25)^. If a food item was not chosen by a participant, a value of 0 was assigned. Furthermore, for all items selected by the participants, the energy content, macronutrients, saturated fatty acids (SFA), sugar, dietary fibre and salt content were calculated based on the chosen amounts. The data were obtained from the Swiss food composition database version 6.4 (https://naehrwertdaten.ch) and from the retailer information. To compare the served amounts of carbohydrates, sugar, fat, SFA, fibre, protein and salt between the two experimental conditions, the percentages of the total energy served from these nutrients were calculated for every individual. The amount of carbohydrates, sugar and protein (in grams) was multiplied by 4 kcal, and the amount of fat and SFA (in grams) was multiplied by 9 kcal (https://www.dge-sh.de/energiegehalt.html). Lastly, the energy served from these nutrients were divided by the total energy content (in kcal) and multiplied by 100.

The food items were further classified into food groups (e.g. vegetables, dairy products and sauces) by summing up the energy content of the individual food items.

### Questionnaire

The questionnaire included self-reported information on demographics, such as sex, age and education, and dieting attempts. Besides the demographic variables, self-reported weight and height were assessed to calculate the participants’ BMI (kg/m^2^). Additionally, the participants rated their hunger status from 1 = *not hungry at all* to 6 = *very hungry*. Overall, the participants were not hungry (M = 2·62, sd = 1·27).

#### Energy needs

The participants’ energy needs (EN) per d (in kilocalories (kcal)) were calculated by multiplying their basal metabolic rates by their physical activity levels. Their basal metabolic rates were calculated based on the Mifflin–St. Jeor equation^(26)^, and their physical activity levels were calculated according to Johansson and Westerterp^(27)^.

#### Perceived tastiness, perceived filling and food choice motives

The questionnaire further included questions about the participants’ specific food choices. The participants rated their perceived tastiness of their food selections in both conditions separately by answering the question ‘How tasty would you find your meal choices?’ from 1 = *not tasty at all to* 5 = *very tasty* (in German: ‘Wie gut würden Ihnen Ihre ausgewählten Mahlzeiten schmecken?’, 1 = *gar nicht schmecken*, 5 = *sehr gut schmecken*). Furthermore, participants rated their perceived filling of their food selections by answering the question ‘How filling would you find your meal choices?’ from 0 = *not at all filling* to 100 = *very filling* (in German: ‘Wie sättigend würden Sie die Auswahl ihrer Mahlzeiten empfinden?’, 0 = *gar nicht sättigend*, 100 = *sehr sättigend*). They were also asked how important each food choice motive was for them when assembling their foods. The included motives were taste, naturalness, product category (e.g. meat, dairy products and fruit), kilocalories, carbohydrate content, sugar content, protein content, fat content, health and familiarity. Each item was rated from 1 = *not important at all* to 6 = *very important.*

### Data analysis

An ANOVA with Bonferroni-adjusted *post hoc* tests was used to assess the difference between EN and total kcal in the ND and the WLD conditions. Furthermore, paired *t* tests were used to evaluate the differences between the two conditions regarding the participants’ tastiness and filling perceptions of their chosen foods. A repeated-measures ANOVA with Bonferroni-adjusted pairwise comparisons was performed to compare the effects of the two conditions (ND and WLD) on the importance of food choice motives.

The assumptions for parametric testing were not fulfilled for the food items and the nutritional properties of the foods. Therefore, Wilcoxon signed-rank tests were used to evaluate the differences between the two study conditions regarding nutrient contents. McNemar tests were performed to determine whether the participants skipped meals in one or the other study condition and to explore which food groups were chosen or not chosen in either of the two study conditions. Analyses were conducted separately by sex. To assess the differences between the sexes, independent *t* tests for tastiness and filling perceptions, as well as for food choice motives, were performed. All statistical analyses were performed using the IBM SPSS version 28.0. A significance level of *P* ≤ 0·05 was used in this study.

## Results

### Energy needs and served kilocalories

**Fig. 3.**
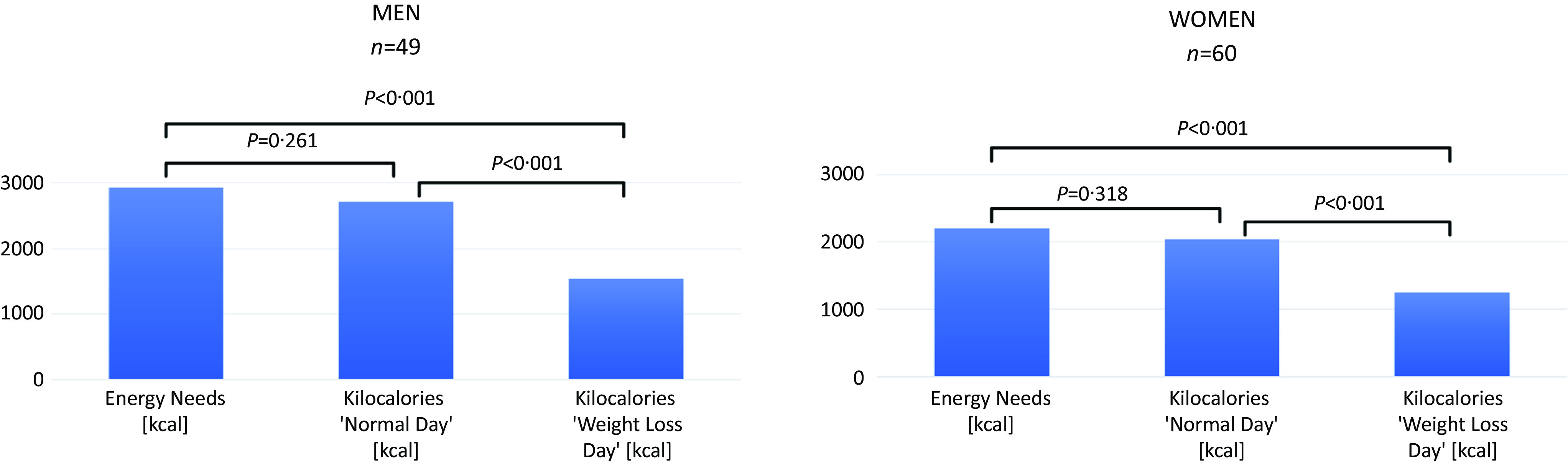
Differences between energy needs, total kilocalories selection for the ‘normal day’ and for the ‘weight loss day,’ separated by sex. *Note*. Two participants did not report their heights and weights; therefore, their energy needs could not be calculated.

An ANOVA showed that the participants’ energy needs (EN) and the total kilocalories (kcal) from the ‘normal day’ (ND) and the ‘weight loss day’ (WLD) food selections differed significantly for men (*F*(2,96) = 84·95, *P* < 0·001, *ω* ^2^ = 0·64) and women (*F*(2,118) = 72·78, *P* < 0·001, ω^2^ = 0·55) (see Fig. 3). *Post hoc* analyses revealed no difference between EN and ND energy selection, neither for men (*P* = 0·261) nor for women (*P* = 0·318). *Post hoc* analyses further revealed a significant difference between EN and WLD, and between ND and WLD for both men (*P* = <0·001) and women (*P* = <0·001). Overall, men’s food selection, on average, amounted to 2704 kcal (sd = 945) in the ND condition and 1537 kcal (sd = 532) in the WLD condition. Women’s food selection, on average, amounted to 2034 kcal (sd = 766) in the ND condition and 1266 kcal (sd = 496) in the WLD condition. Moreover, 95 % of the participants selected a WLD food composition whose total kcal were below their EN. In other words, the participants selected approximately as many kcal for the ND and fewer kcal for the WLD as they physiologically would need to maintain their current weights. However, on average, men reduced the energy by 1166 kcal (sd = 936), while women reduced the energy by 768 kcal (sd = 667) in the WLD food selection compared with the ND food selection.

### Meal skipping and meal ratings

When comparing the main meals and snack frequencies between the two study conditions (ND *v*. WLD), McNemar tests showed that women were significantly more likely to skip their snacks on the WLD compared with the ND *(P* = 0·04, OR = 29·38). In particular, eight women chose a snack for the ND but no snack for the WLD. Five men skipped their snacks for the WLD, although they chose a snack for the ND (*P* > 0·05). There were no significant main meal skips for both men and women. In other words, men and women chose the same main meals (breakfast, lunch and dinner) for the ND as for the WLD.

The participants further indicated their perceived tastiness and perceived filling of their food selection in both conditions separately. They perceived their WLD food selection as significantly less tasty (M = 4·6, sd = 1, *P* < 0·001, Cohen’s |*d*|= 0·56) and less filling (M = 66, sd = 20, *P* < 0·001, Cohen’s |*d*|= 0·88) compared with their ND food selection (tastiness: M = 4·2, sd = 1, filling: M = 83, sd = 16). Nevertheless, women found their WLD food selection (M = 4·3, sd = 1) significantly tastier than men did (M = 4·0, sd = 1, *P* = 0·04, Cohen’s |*d*| = 0·40).

### Dietary weight loss strategies

Table 2 (men) and Table 3 (women) provide insights into the differences in food selection between the ND and the WLD conditions. Table 4 displays the changes in kcal by food group between the ND and the WLD. Detailed information on which food items are classified under which food groups can be found in the supplementary material.

**Table 2.** Food group selection for the ‘normal day’ and the ‘weight loss day’ for men (*n* 50) and corresponding McNemar tests

		Weight loss day					
		Not taken		Taken		McNemar	
	Normal day	*n*	%	*n*	%	*P*	OR
Fruits	Not taken	3	30	7	70	0·180	8·14
	Taken	2	5	38	95		
Vegetables	Not taken	0	0	2	100	–*	–*
	Taken	0	0	48	100		
Pulses	Not taken	12	50	12	50	0·359	2·71
	Taken	7	27	19	73		
Unsweetened beverages	Not taken	0	0	2	100	1·00	1·02
	Taken	1	2	47	98		
SSB	Not taken	29	94	2	7	0·007	6·69
	Taken	13	68	6	32		
Zero beverages	Not taken	34	92	3	8	0·508	13·22
	Taken	6	46	7	54		
Bread and croissants	Not taken	5	83	1	17	0·002	11·92
	Taken	13	30	31	71		
Cereals	Not taken	21	78	6	22	0·454	4·56
	Taken	10	44	13	57		
Margarine and butter	Not taken	30	91	3	9	0·021	3·08
	Taken	13	77	4	24		
Sauce	Not taken	0	0	1	100	0·003	1·32
	Taken	12	25	37	76		
Eggs	Not taken	21	72	8	28	0·503	1·97
	Taken	12	57	9	43		
Dairy products	Not taken	0	0	1	100	0·375	1·09
	Taken	4	8	45	92		
Total meat	Not taken	8	67	4	33	0·007	2·47
	Taken	17	45	21	55		
Poultry	Not taken	23	62	14	38	0·286	1·03
	Taken	8	62	5	39		
Unprocessed red meat	Not taken	30	97	1	3	0·003	17·50
	Taken	12	63	7	37		
Processed meat	Not taken	30	100	0	0	< 0·001	1·11
	Taken	18	90	2	10		
Fish and fish sticks	Not taken	25	69	11	31	0·824	1·26
	Taken	9	64	5	36		
Pasta, potato and rice	Not taken	2	25	6	75	0·167	0·74
	Taken	13	31	29	69		
Fast food	Not taken	27	93	2	7	< 0·001	1·42
	Taken	19	91	2	10		
Vegetarian alternatives	Not taken	31	80	8	21	0·227	10·33
	Taken	3	27	8	73		
Salty snacks	Not taken	18	75	6	25	0·006	0·71
	Taken	21	81	5	19		
Sweets	Not taken	12	92	1	8	< 0·001	19·71
	Taken	14	38	23	62		

SSB, sugar-sweetened beverages.

* No test statistics can be calculated if two cells are empty.

**Table 3.** Food group selection for the ‘normal day’ and the ‘weight loss day’ for women (*n* 61) and corresponding McNemar tests

		Weight loss day					
		Not taken		Taken		McNemar	
	Normal day	*n*	%	*n*	%	*P*	OR
Fruits	Not taken	1	20	4	80	1·00	3·25
	Taken	4	7	52	93		
Vegetables	Not taken	0	0	4	1	0·687	1·04
	Taken	2	4	55	97		
Pulses	Not taken	13	57	10	44	0·678	2·50
	Taken	13	34	25	66		
Unsweetened beverages	Not taken	0	0	1	100	–*	–*
	Taken	0	0	60	100		
SSB	Not taken	49	100	0	0	0·004	1·33
	Taken	9	75	3	25		
Zero beverages	Not taken	40	93	3	7	0·344	20·95
	Taken	7	39	11	61		
Bread and croissants	Not taken	17	77	5	23	0·096	6·80
	Taken	13	33	26	67		
Cereals	Not taken	30	81	7	19	0·804	7·14
	Taken	9	38	15	63		
Margarine and butter	Not taken	42	93	3	7	0·035	4·67
	Taken	12	75	4	25		
Sauce	Not taken	3	43	4	57	0·049	2·37
	Taken	13	24	41	76		
Eggs	Not taken	20	56	16	44	1·00	0·83
	Taken	15	60	10	40		
Dairy products	Not taken	1	50	1	50	0·219	10·80
	Taken	5	9	54	92		
Total meat	Not taken	19	68	9	32	0·523	3·25
	Taken	13	39	20	61		
Poultry	Not taken	29	62	18	38	0·043	1·61
	Taken	7	50	7	50		
Unprocessed red meat	Not taken	42	91	4	9	0·118	3·82
	Taken	11	73	4	27		
Processed meat	Not taken	47	98	1	2	0·003	3·92
	Taken	12	92	1	8		
Fish and fish sticks	Not taken	33	75	11	25	0·839	0·92
	Taken	13	77	4	24		
Pasta, potato and rice	Not taken	6	46	7	54	0·064	1·56
	Taken	17	35	31	65		
Fast food	Not taken	46	100	0	0	< 0·001	1·07
	Taken	14	93	1	7		
Vegetarian alternatives	Not taken	37	80	9	20	1·00	2·74
	Taken	9	60	6	40		
Salty snacks	Not taken	25	78	7	22	0·189	3·83
	Taken	14	48	15	52		
Sweets	Not taken	13	93	1	7	< 0·001	6·71
	Taken	31	66	16	34		

SSB, sugar-sweetened beverages.

* No test statistics can be calculated if two cells are empty.

**Table 4.** Comparisons of food groups’ kilocalories between the ‘normal day’ and the ‘weight loss day’, and Wilcoxon signed-rank tests, separated by sex

	Normal day		Weight loss day		Wilcoxon signed-rank test		
Kilocalories	Mdn	IQR	Mdn	IQR	*Z*	*P*	*|r|*
Men (*n* 50)							
Fruits	126	71–328	210	95–361	–1·34	0·181	0·19
Vegetables	51	25–99	64	34–134	–2·18	0·029	0·31
Pulses	4	0–50	18	0–60	–0·84	0·402	0·12
Unsweetened beverages (ml)	944	500–1400	1075	694–1613	–2·32	0·020	0·33
SSB	0	0–185	0	0–0	3·12	0·002	0·44
Zero beverages (ml)	0	0–450	0	0–0	–1·46	0·145	0·21
Bread and croissants	210	126–407	72	0–144	4·79	< 0·001	0·68
Cereals	0	0–221	0	0–219	0·69	0·493	0·10
Margarine and butter	0	0–110	0	0–0	2·89	0·004	0·41
Sauce	182	111–329	108	4–172	4·30	< 0·001	0·61
Eggs	0	0–78	0	0–78	0·77	0·442	0·11
Dairy products	394	233–574	254	159–411	3·61	< 0·001	0·51
Total meat	163	12–313	128	0–227	1·26	0·208	0·18
Poultry	0	0–68	128	0–204	–4·07	< 0·001	0·58
Unprocessed red meat	0	0–80	0	0–0	3·10	0·002	0·44
Processed meat	0	0–224	0	0–0	3·71	< 0·001	0·52
Fish and fish sticks	0	0–204	0	0–204	–0·08	0·934	0·01
Pasta, potato and rice	128	25–186	31	0–81	3·45	< 0·001	0·49
Fast food	0	0–494	0	0–0	3·72	< 0·001	0·53
Vegetarian alternatives	0	0–0	0	0–22	–1·03	0·305	0·15
Salty snacks	166	0–332	0	0–0	2·63	0·009	0·37
Sweets	180	0–316	0	0–82	4·87	< 0·001	0·68
							
Women (*n* 61)							
Fruits	167	80–308	197	102–266	–0·40	0·691	0·05
Vegetables	48	22–68	60	35–88	–2·64	0·008	0·34
Pulses	11	0–54	9	0–54	0·67	0·505	0·09
Unsweetened beverages (ml)	1000	512–1400	1150	725–1500	–1·73	0·084	0·22
SSB	0	0–0	0	0–0	2·81	0·005	0·36
Zero beverages (ml)	0	0–450	0	0–0	2·01	0·044	0·26
Bread and croissants	128	0–245	38	0–93	4·38	< 0·001	0·56
Cereals	0	0–221	0	0–219	0·55	0·584	0·07
Margarine and butter	0	0–40	0	0–0	2·72	0·007	0·35
Sauce	151	77–310	113	0–218	3·43	< 0·001	0·44
Eggs	0	0–78	0	0–78	–0·67	0·500	0·09
Dairy products	315	192–553	273	92–381	2·85	0·004	0·36
Total meat	33	0–175	102	0–204	–0·90	0·369	0·12
Poultry	0	0–0	74	0–189	–3·73	< 0·001	0·48
Unprocessed red meat	0	0–8	0	0–0	0·36	0·717	0·05
Processed meat	0	0–0	0	0–0	2·85	0·004	0·36
Fish and fish sticks	0	0–128	0	0–51	0·12	0·904	0·02
Pasta, potato and rice	38	1–134	22	0–65	2·04	0·041	0·26
Fast food	0	0–62	0	0–0	3·41	< 0·001	0·44
Vegetarian alternatives	0	0–5	0	0–7	–0·20	0·841	0·03
Salty snacks	0	0–319	0	0–231	3·22	0·001	0·2041
Sweets	171	38–336	0	0–26	5·84	< 0·001	0·75

SSB, sugar-sweetened beverages.

Mdn and IQR indicate only 50 % and 25–75 % of the values. However, some products were selected only by a few people; therefore, zero values can occur and still result in significant test results.

McNemar tests showed that on a product level (taken *v*. not taken, independent of amount), there were significant differences between the ND and the WLD for women and men. A significant number of men and women omitted SSB, margarine and butter, sauce, processed meat, fast food, and sweets (*P* < 0·05) for weight loss purposes, meaning that they chose these food groups for the ND food selection but did not choose them for the WLD food selection. Men additionally omitted bread and croissants (*P* = 0·002), red meat (*P* = 0·003), and salty snacks (*P* = 0·02) for the WLD but chose them for the ND. An increase in poultry consumption was observed among women. Of the women who did not choose poultry for the ND, 38 % did so for the WLD. No significant effects were found for vegetables, fruits and pulses for either man or woman.

The analyses of energy changes within food groups showed that both men and women significantly selected more kcal from vegetables in the WLD condition. Men also significantly increased the amount (in millilitres) of unsweetened beverages (*P* = 0·02) and the kcal from poultry (*P* < 0·001) for weight loss purposes. Additionally, the kcal were significantly decreased for dairy products, pasta, rice and potato, and salty snacks for the WLD food selection compared with that for the ND in both sexes. Women further selected significantly less millilitres from zero beverages (*P* = 0·04) and fewer kcal from bread and croissants (*P* < 0·001). There were no statistically significant changes in the selection of kcal from fruits, pulses, cereals, eggs, fish and fish sticks, and vegetarian alternatives (*P* > 0·05) in the ND and the WLD conditions.

### Importance of food choice motives

**Fig. 4.**
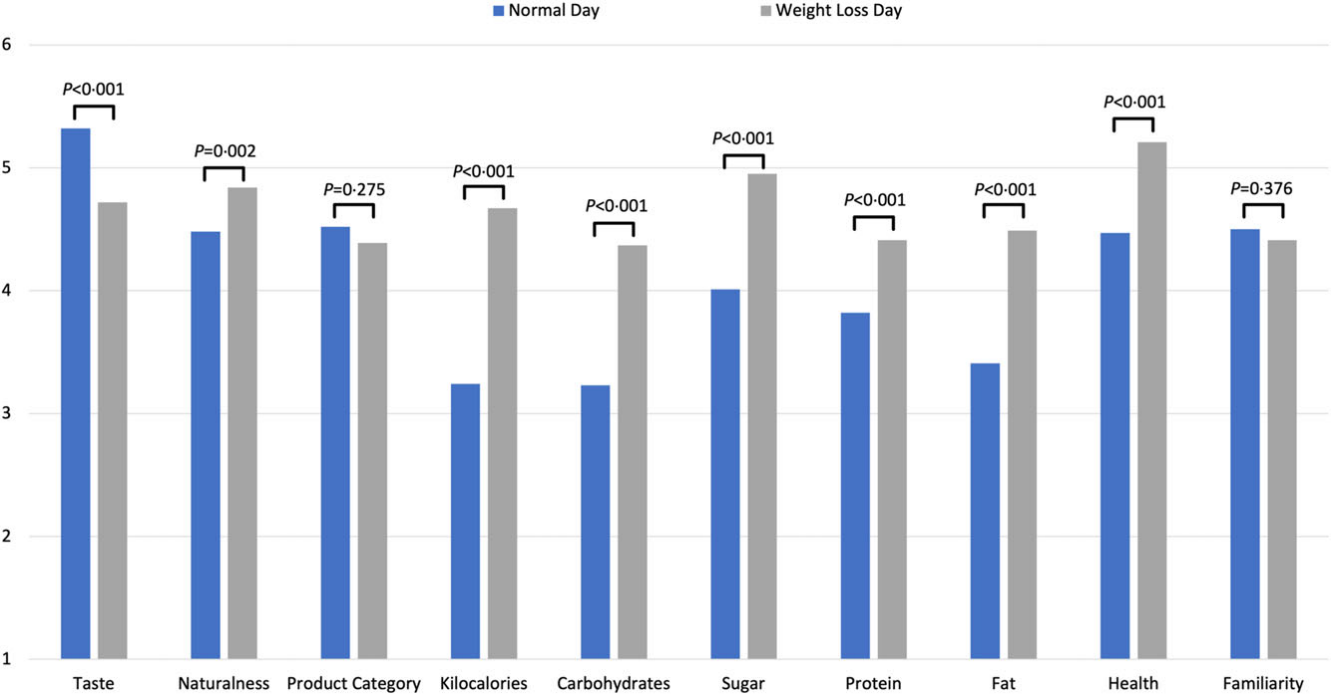
Motives important for food choice. *Note.* Each motive was rated separately for each condition (ND and WLD) on a scale ranging from 1 = *not important at all* to 6 = *very important*. Means for every food choice motive is displayed separately for the ND and the WLD condition, and *P*-values from ANOVA with Bonferroni-adjusted *post hoc* tests are shown.

A repeated-measures ANOVA with Bonferroni-adjusted pairwise comparisons was performed to compare the effects of the two conditions (ND and WLD) on the importance of food choice motives. There was a significant interaction effect between the conditions and the importance of food choice motives (*F*(9, 990) = 31·68, *P* < 0·001, η_p_^2^ = 0·22). Pairwise comparisons showed that taste was significantly less important for the WLD food selection than that for the ND (*M*_*normal*_ = 5·32, se = 0·90, *M*_*weight loss*_ = 4·72, se = 1·03, *F*(1, 110) = 32·11, *P* < 0·001, η_p_^2^ = 0·23). In contrast, naturalness, kilocalories, carbohydrate content, sugar content, protein content, fat content and health were significantly more important for the WLD food selection than that for the ND (*P* < 0·01). There was no difference between ND and WLD regarding the importance of the product category and familiarity (*P* > 0·05) for food choice (see Fig. 4).

For their food choices, men assigned less importance to health (*P* = 0·03, Cohen’s |*d*| = 0·43), which accounted for the only sex difference for the ND selection. For the WLD selection, taste (*P* = 0·002, Cohen’s |*d*| = 0·60), naturalness (*P* = 0·01, Cohen’s |*d*| = 0·50) and health (*P* = 0·004, Cohen’s |*d*| = 0·56) were more important food choice motives for women than for men.

### Proportion of nutrients and dietary recommendations

Differences in nutrient relations between the ND condition and the WLD condition are summarised in Table 5. In the WLD condition, the participants served themselves a significantly higher percentage of total energy from protein and sugar, while choosing a lower percentage of total energy from total fat and saturated fatty acids (SFA).

**Table 5.** Nutrient comparison between the ‘normal day’ and the ‘weight loss day’, and Swiss dietary recommendations, separated by sex

	Normal day		Weight loss day		Wilcoxon signed-rank test			Dietary recommendations
	Mdn	IQR	Mdn	IQR	Z	*P*	*|r|*	
Men (*n* 50)								
Carbohydrates (% of TE)	41	35–45	43	36–50	–1·55	0·121	0·22	45–55 % TE^‡^
Sugar (% of TE)	19	15–25	23	17–31	–3·46	< 0·001	0·49	< 10 % TE^†^^,^^‡^
Total fat (% of TE)	41	37–45	34	29–39	3·67	< 0·001	0·52	< 30 % TE*,^†^,^‡^
SFA (% of TE)	15	12–19	12	7–16	1·97	0·048	0·28	<10 % TE*,^†^,^‡^
Protein (% of TE)	16	14–19	19	16–24	–4·10	< 0·001	0·58	10–20 % TE^‡^
Fibre (g)	26	21–37	24	17–33	2·09	0·037	0·30	> 30 g/day*
Salt (g)	8	6–11	6	4–7	4·82	< 0·001	0·68	< 5 g/day^†^
								
Women (*n* 61)								
Carbohydrates (% of TE)	39	33–44	38	32–43	0·76	0·449	0·10	45–55 % TE^‡^
Sugar (% of TE)	20	17–26	23	18–29	–1·78	0·075	0·23	< 10 % TE^†^,^‡^
Total fat (% of TE)	41	36–46	37	27–44	4·05	< 0·001	0·52	< 30 % TE*,^†^,^‡^
SFA (% of TE)	16	13–19	12	8–16	4·43	< 0·001	0·57	< 10 % TE*,^†^,^‡^
Protein (% of TE)	17	13–21	23	15–29	–4·62	< 0·001	0·59	10–20 % TE^‡^
Fibre (g)	24	15–29	22	16–28	2·99	0·003	0·38	> 30 g/day*
Salt (g)	6	4–8	4	3–6	4·69	< 0·001	0·60	< 5 g/day^†^

TE, total energy served; SFA, saturated fatty acids.

* German Society for Nutrition (DGE) reference values^(41)^.

† WHO recommendations^(42)^.

‡ Sixth Swiss Nutrition Report^(40)^.

As shown in Table 5, the average nutrient selection for both days deviated from the Swiss dietary recommendations for both sexes. The recommended intake levels for carbohydrates and fibre were not reached on either day, whereas the portions for sugar, total fat and SFA exceeded the recommendations on either day. The recommended protein intake levels were met or were even slightly exceeded on both days and for both sexes. The salt intake recommendations for women were only exceeded on the ND food selection, while those for men were exceeded on both days.

## Discussion

The goal of the present study was to investigate consumers’ ability to select foods for an entire day when imagining their desire to lose weight. The study further examined whether their choices followed the official dietary recommendations and what food choice motives drove their overall selections. The results indicated that the participants composed meals for the entire day that provided less energy than their daily energy needds (EN). However, energy reduction was on average very high (1166 kcal and 768 kcal, for men and women, respectively). The weight loss strategies that were used consisted of increasing the amounts of vegetables and unsweetened beverages (only men), while reducing high-fat and high-energy products. Dietary recommendations were not met in both the ‘normal day’ (ND) and the ‘weight loss day’ (WLD) conditions. Health, kilocalories and nutrient content were more important food choice motives for a WLD than for an ND.

Generally, participants’ energy selection for the ND condition was almost the same as their calculated EN. This means that the participants were able to meet their EN for a full day quite well. Considering that neither their EN nor the kilocalories of the food items were displayed to them, this is surprising. It shows that people are capable of making food choices to maintain their weight. However, compared with the ND, participants reduced their energy selection on average up to 768 kcal (women) and 1166 kcal (men) per day on the WLD. Given that a daily calorie reduction of 500 kcal leads roughly to a weight reduction of up to 0·5 kg per week^(28 and 29)^, the female participants of this study would roughly lose up to 0·7 kilogram body weight per week and the male participants roughly up to 1·1 kilogram body weight per week if they maintained the calorie deficit on a daily basis. This is a quite high energy deficit and body weight reduction^(30)^ and a potential problem, as a large energy deficit will likely make participants less compliant in their weight control behaviours over the long term. However, unplanned in-between consumption is common, especially when people keep their energy intake on a low level. Therefore, the actual energy deficit will probably be smaller than the one found in this study, since no unplanned in-between consumption could be measured.

### Dietary weight loss strategies

Different dietary weight loss strategies have been identified in previous research through questionnaires and interviews. A common strategy involves individuals’ avoidance or restricted intake of specific foods (for a review, see Santos *et al.* ^(3)^), such as desserts, SSB and fried foods^(10)^, consistent with the present study’s results. This study’s participants decreased or even avoided the selection of high-fat and high-energy products, including sauces (e.g. cream, carbonara and gravy), fast food (e.g. cheeseburger and pizza), sweets and sugar-sweetened beverages (SSB) for a WLD compared with a ND. Although some participants omitted sweets entirely for weight loss purposes, sweets did not seem to be replaced with fruits, since no change in the amount of fruits selected was observed. Therefore, reducing high-fat and high-energy foods seems to be an energy-saving strategy that people know and implement.

A further strategy used by both sexes involved increasing the amount of vegetables in the WLD condition compared with the ND condition. Previous studies have shown that increased consumption of vegetables helps people lose weight^(8 and 31)^ because vegetables have a low energy density, contain essential vitamins and minerals, and are sources of water and fibre^(31–33)^. Therefore, increasing the amount of vegetables is a key strategy to facilitate weight loss, which was employed by the participants of the present study.

Regarding the consumption of animal protein, the results showed no change in the total meat consumption between the study conditions, neither for men nor for women. Only for the subcategories *processed meat and poultry*, less of the first one and more of the second one was selected in the WLD condition. This strategy seems reasonable, as poultry is one of the leanest types of meat, with high-protein, low-fat and low-energy contents^(34)^. In contrast, processed meat generally has the highest fat content of up to 25 %^(35)^. Therefore, decreasing processed meat consumption and increasing poultry consumption could facilitate weight loss by reducing energy intake, which was a strategy applied by the participants of the present study in a simulated setting. Fish is another source of animal protein, is part of a healthy diet and is suggested to support weight reduction^(10 and 36 and 37)^. However, the participants of the present study did not select more fish in the WLD condition, and only a few of them selected it in the ND condition. A possible explanation is the generally low level of fish consumption in the German-speaking part of Switzerland^(38)^. Alternative plant-based protein sources are pulses (e.g. peas and green beans). The participants selected pulses in both conditions but did not increase the portion size in either condition. This finding seems to indicate the need to raise awareness about pulses^(39)^ and fish as substitutes for meat and the importance of reducing high amounts of processed meat to facilitate weight loss.

Decreasing or avoiding the intake of SSB is a further way to limit energy intake by decreasing the sugar intake. Furthermore, drinking a lot of water is a diet strategy too as it has been suggested to suppress hunger pangs and therefore minimise energy intake, especially if SSB are replaced by water^(15)^. In the present study, men used these weight loss strategies by selecting more unsweetened beverages and omitting SSB in the WLD condition. However, this result contradicts another study’s finding that women reported drinking a lot of water more often than men did^(15)^. However, in the current study, women chose a higher volume of unsweetened beverages than men did, regardless of the condition. Although the effectiveness of water as a strategy is unknown^(15)^, switching from SSB to unsweetened beverages is an easily implementable strategy to decrease energy intake by decreasing sugar intakes.

Overall, participants were aware that avoiding SSB and high-energy foods while increasing the consumption of vegetables and unsweetened beverages are reasonable dietary changes to lose weight. Moreover, these strategies are also in line with public nutrition recommendations^(29,40)^. Therefore, further efforts should be made to help the population make favourable dietary changes and maintaining them, not only to lose weight but also to maintain a healthy weight.

### Dietary recommendations

The participants’ food selection for weight loss had a lower percentage of total energy from total fat and saturated fatty acids (SFA) but a higher percentage of total energy from protein. Except for protein, participants’ choices differed from the Swiss dietary recommendations in both conditions. Moreover, the percentage of total energy from carbohydrates was not altered between the study conditions and was lower than the Swiss nutrition recommendations^(40)^. This lower amount of carbohydrates could reflect the popularity of low-carbohydrate diets, even in normal eating conditions^(25)^.

Participants fibre quantity differed from the recommended quantity of > 30 g per day^(40–42)^ in either condition. This is in line with previous studies showing that the fibre content in meals was too low^(25 and 43)^. Dietary fibres have been shown to facilitate weight loss^(8)^ by increasing satiety^(44)^, and they provide various health benefits, such as lowering the risks for diabetes, obesity or coronary heart diseases^(45)^. However, the present study’s participants did not choose more pulses for weight loss purposes, which are high in fibre and protein and low in fat^(46)^. Therefore, it is important to raise more awareness about the benefits of pulses and vegetables and how they can be integrated into daily meals.

Even though the participants reduced their total fat intake in this experimental setting, and the study neglected food preparation (e.g. frying with oil or butter), the amount was still above Swiss recommendations^(40)^. The participants chose more SFA in both conditions than recommended, confirming the findings of a previous study^(25)^. However, reducing fat intake helps to lose weight by decreasing the total energy intake, as fat has double the number of kcal for the same amount than carbohydrates or proteins have^(6)^. Thus, the participants of the current study seemed to understand that they should reduce their fat intake to decrease their energy consumption. However, there seems to be a lack of knowledge about how much fat and especially how much SFA different food items have. Therefore, greater awareness about the fat content of different food items should be promoted, not only for weight loss purposes.

Overall, reducing energy intake to a minimum and avoiding or restricting the consumption of single food groups are insufficient strategies, since long-term adherence to this dietary regime will probably be low. Instead, it is necessary to modify people’s diets in a sustainable way, especially to comply with dietary guidelines.

### Food choice motives

Besides the dietary strategies, this study’s results show that if people are asked to put together meals for an entire day to lose weight (WLD), taste as a food choice motive becomes less important to them compared with an ND composition. Perceived tastiness also decreased for the weight loss meals compared with the ND meals, even though the participants could choose whatever they liked. Nonetheless, taste is generally the most important food choice motive^(19)^; therefore, sacrificing taste for weight loss purposes could lead to compliance problems, which might result in relapses into old dietary behaviour patterns over time.

The more important food choice motives for weight loss purposes were kilocalories and nutrient contents (carbohydrates, sugar, protein and fat). This partially supports a previous study’s finding that nutrition information was more important for food decision-making by people with a weight loss intention than for people without that intention^(47)^. It is reasonable that people focus more on kilocalories and nutrient contents of various foods when trying to lose weight since they must attain und maintain a negative energy balance.

### Limitations

The participants were required to select foods for an entire day; however, it is unclear whether they would have eaten everything they chose or would have consumed even more in reality. Furthermore, by using a food buffet, the experimental setting was highly controlled and did not take into account other factors, such as price, cooking skills or food availability, which are important determinants for food choice^(48)^. Since food preparation (frying and seasoning) was not considered in this study, underestimations, especially of fat and salt intake, cannot be ruled out. However, the participants’ energy selection for the ND was almost the same as their calculated EN. Therefore, their choices seemed to represent good guesses of their daily energy intake.

The authors were interested in the participants’ food selections and the changes in their food selections for weight loss. This was a hypothetical WLD and did not show food selections over a longer period. Furthermore, the food buffet excluded alcoholic beverages, which is a further limitation of this study. However, the authors decided not to offer alcoholic beverages based on the assumption that the participants would not drink alcohol daily but rather in social situations. Alcohol is also highly susceptible to underreporting^(49)^, which would have affected this study’s results. Furthermore, the direction of the importance of each food choice factor in this study is not clear. For example, it could be that participants thought it was important to reduce carbohydrates to lose weight, but it could also be that they thought it was important to increase carbohydrates to lose weight. Therefore, further research is needed to identify the direction of the importance of different food choice motives.

It is easier for people to reduce their daily energy intake than to fundamentally increase their physical activities in daily life. Furthermore, most individuals fail to achieve adequate energy expenditure through exercise, or they even compensate for the energy burned by eating afterwards^(50)^. Thus, the current study focused on food selection and neglected physical activity. The authors cannot rule out the possibility of the participants’ attempts to compensate for their lack of physical activity (i.e. not included as a strategy) by an even higher reduction in their food selection.

Compared with the general Swiss population^(51–53)^, our study had more participants with a higher educational level (65 % *v*. 45 %) and included more people with a normal BMI range of 18·5–24·9 kg/m^2^ (62 % *v*. 55 %). This may have had an impact on the results of this study; thus, the results should be interpreted with caution.

### Conclusion

This study examined the dietary strategies that people would tend to implement in a real-life decision-making food choice situation for weight loss purposes and the motives behind their food selections. The results suggest that people use the appropriate strategy of increasing their selection of low-energy products (e.g. vegetables) while decreasing their choice of high-energy and high-fat products (e.g. sweets). Furthermore, their food selections for weight loss are based mostly on their food choice motives – taste, kilocalories and nutrient content. Therefore, this study shows that people are capable of choosing an adequate number of kilocalories to maintain their body weights, as well as implementing a lower energy intake for weight loss. While reducing energy intake to a great extent, they would probably still consume too much fat and sugar, as well as too little carbohydrates and fibres, and taste would become less important. As a result, the new diet would more likely be abandoned over the long term, leading to a weight regain. However, it is encouraging that the participants knew how to make appropriate dietary changes to lose weight. Further studies should examine strategies that can support consumers in implementing and sustaining these dietary modifications over an extended period.

## Supporting information

Giacone et al. supplementary material Giacone et al. supplementary material

## Supplementary material

For supplementary material/s referred to in this article, please visit https://doi.org/10.1017/S0007114523002726
