# Supplementary material for: Food choices for weight loss: what dietary strategies would people use?
Source: Br J Nutr. 2023 Nov 28;131(7):1268–80. doi: 10.1017/S0007114523002726 (PMC10918523; doi:10.1017/S0007114523002726)
Supplement: Giacone et al. supplementary material [file S0007114523002726sup001.pdf]

Supplementary Material

Details of the 152 food items used in the present study (from A to Z).

Food Item	Portion Size	Real (R) or Fake Food (F)	Food Group
7up[1]	500 ml	R	SSB
7up Zero[1]	500 ml	R	Zero
Apple	140 g	F	Fruit
Apple Puree	260 g	F	Fruit
Apple Tarte	132 g	F	Sweets
Bacon	10 g	F	Unprocessed Red Meat
Banana	126 g	F	Fruit
Basil Pesto (large)	118 ml	F	Sauce
Basil Pesto (medium)	76 ml	F	Sauce
Basil Pesto (small)	25 ml	F	Sauce
Bell Pepper	Continuous	F	Vegetables
Blackberry	4 g	F	Fruit
Boiled Egg	50 g	F	Eggs
Bolognese Sauce (large)	118 ml	F	Sauce
Bolognese Sauce (medium)	76 ml	F	Sauce
Bolognese Sauce (small)	25 ml	F	Sauce
Bread Roll White	50 g	F	Bread
Bresaola	10 g	F	Unprocessed Red Meat
Brie Cheese	26 g	F	Milk Products
Broccoli	Continuous	F	Vegetables
Butter	15 g	R	Butter & Margarine
Cappuccino	150 ml	F	Milk Products
Carbonara Sauce (large)	118 ml	F	Sauce
Carbonara Sauce (medium)	76 ml	F	Sauce
Carbonara Sauce (small)	25 ml	F	Sauce
Carrot	Continuous	F	Vegetables
Carrot Cake	104 g	F	Sweets
Cauliflower	Continuous	F	Vegetables
Cheese Cube	9 g	F	Milk Products
Cheese Stripe	18 g	F	Milk Products
Cheese Tarte	135.6 g	F	Salty Snacks
Cheeseburger	220.91 g	F	Fast Food
Cherry	7 g	F	Fruit
Chicken Breast	100 g	F	White Meat
Chicken Nuggets	27.8 g	F	Processed Meat
Chicken Strips	Continuous	F	White Meat
Chips Nature	30 g	R	Salty Snacks

Food Item	Portion Size	Real (R) or Fake Food (F)	Food Group
Chips Paprika	30 g	R	Salty Snacks
Choco Pops (Kellogs)	30 g	F	Cereals
Chocolate Cake	104 g	F	Sweets
Chocolate Drink	250 ml	R	Milk Products
Chocolate Drink High Protein	330 ml	R	Milk Products
Chocolate Marshmallow	23 g	F	Sweets
Coca Cola	450 ml	R	SSB
Coca Cola Zero	450 ml	R	Zero
Coffee	150 ml	F	Unsweetened Beverages
Coffee Cream	12 ml	R	Milk Products
Cookies	8 g	F	Sweets
Corn	Continuous	F	Vegetables
Corn Flakes (Kellogs)	20 g	R	Cereals
Cottage Cheese	200 g	R	Milk Products
Cream Sauce (large)	118 ml	F	Sauce
Cream Sauce (medium)	76 ml	F	Sauce
Cream Sauce (small)	25 ml	F	Sauce
Cream Sauce with Mushrooms (large)	118 ml	F	Sauce
Cream Sauce with Mushrooms (medium)	76 ml	F	Sauce
Cream Sauce with Mushrooms (small)	25 ml	F	Sauce
Crispbread	11.4 g	F	Bread
Croissant Butter	45 g	F	Bread
Croissant Silser	90 g	F	Bread
Cucumber Slice	5.1 g	F	Vegetables
Curry Sauce (large)	118 ml	F	Sauce
Curry Sauce (medium)	76 ml	F	Sauce
Curry Sauce (small)	25 ml	F	Sauce
Danish Pastry	110 g	F	Sweets
Dark Bread Slice	30.6 g	F	Bread
Dark Chocolate	17 g	F	Sweets
Escalope	60 g	F	Unprocessed Red Meat
Espresso	25 ml	F	Unsweetened Beverages
Espresso Zero (Emmi)	230 ml	R	Zero
Falafel	25 g	F	Vegetarian Alternative
Fish Fillet	140 g	F	Fish
Fish Sticks	30 g	F	Fish
Fol Epi Cheese Slice	22 g	F	Milk Products

Food Item	Portion Size	Real (R) or Fake Food (F)	Food Group
French Dressing	60 ml	R	Sauce
French Fries	Continuous	F	Fast Food
Fried Egg	50 g	F	Eggs
Fried Potatoes	Continuous	F	Pasta, Potato & Rice
Frosties (Kellogs)	25 g	R	Cereals
Gravy Sauce (large)	118 ml	F	Sauce
Gravy Sauce (medium)	76 ml	F	Sauce
Gravy Sauce (small)	25 ml	F	Sauce
Green Beans	Continuous	F	Pulses
Herbal Tea	200 ml	F	Coffee, Tea & Water
Honey	25 g	R	Sweets
Honey Pops (Kellogs)	25 g	R	Cereals
Ice Tea Lemon	500 ml	R	SSB
Ice Tea Lemon Zero	500 ml	R	Zero
Ice Tea Peach	500 ml	R	SSB
Ice Tea Peach Zero	500 ml	R	Zero
Italian Dressing	60 ml	R	Sauce
Jam	25 g	R	Sweets
Jam Light	25 g	R	Sweets
Ketchup	35 g	R	Sauce
Kidney Beans	Continuous	F	Pulses
Low-Fat Curd	250 g	R	Milk Products
Macchiato Coffee (Emmi)	230 ml	R	SSB
Mango	350 g	F	Fruit
Margarine	10 g	R	Butter & Margarine
Mayonnaise	35 g	R	Sauce
Meatball	90 g	F	Processed Meat
Meatloaf	74 g	F	Processed Meat
Milk Chocolate	17 g	F	Sweets
Mozzarella	31.14 g	F	Milk Products
Muesli Chocolate	50 g	R	Cereals
Muesli Energy Plus	50 g	R	Cereals
Muesli High Protein	50 g	R	Cereals
Multivitamin Juice	330 g	R	Fruit
Mustard	35 g	R	Sauce
MyMuesli Berry White Chocolate	85 g	R	Cereals
Nut Mix	50 g	R	Salty Snacks
Nut Mix with Raisins	50 g	R	Salty Snacks
Nutella	15 g	R	Sweets

Food Item	Portion Size	Real (R) or Fake Food (F)	Food Group
Orange Juice	330 ml	R	Fruit
Orangina[2]	500 ml	R	SSB
Orangina Zero[2]	500 ml	R	Zero
Peas	Continuous	F	Pulses
Pear	140 g	F	Fruit
Penne White	Continuous	F	Pasta, Potato & Rice
Penne Wholegrain	Continuous	F	Pasta, Potato & Rice
Pizza Slice	105 g	F	Fast Food
Potato Soup	250 ml	F	Pasta, Potato & Rice
Potatoes Steamed	Continuous	F	Pasta, Potato & Rice
Praline	10 g	F	Sweets
Raspberry	6 g	F	Fruit
Redbull	250 ml	R	SSB
Redbull Sugarfree	250 ml	R	Zero
Rice	Continuous	F	Pasta, Potato & Rice
Rivella Blue[3]	500 ml	R	Zero
Rivella Red[3]	500 ml	R	SSB
Roastbeef	50 g	F	Unprocessed Red Meat
Salad	2.5 g	F	Vegetables
Salami Slice	11 g	F	Processed Meat
Sausage	140 g	F	Processed Meat
Schnitzel Breaded	120 g	F	Processed Meat
Schnitzel Sandwich	230 g	F	Fast Food
Smoothie Green	250 ml	R	Fruit
Smoothie Pink	250 ml	R	Fruit
Soy Sauce	10 ml	R	Sauce
Sparkling Water	500 ml	R	Unsweetened Beverages
Spinach Tarte	135.6 g	F	Salty Snacks
Strawberry	16 g	F	Fruit
Strawberry Cake	140 g	F	Sweets
Strawberry Tartlet	120 g	F	Sweets
Sugar Cubes	4 g	F	Sweets
Tap Water	500 ml	R	Unsweetened Beverages
Tartar Sauce	35 g	F	Sauce
Toast	25 g	F	Bread
Tofu Cubes	Continuous	F	Vegetarian Alternative
Tomato Sauce (large)	118 ml	F	Sauce
Tomato Sauce (medium)	76 ml	F	Sauce
Tomato Sauce (small)	25 ml	F	Sauce

Food Item	Portion Size	Real (R) or Fake Food (F)	Food Group
Tomato Slice	12.2 g	F	Vegetables
Tomato Soup	250 ml	F	Vegetables
Veal Chop	140 g	F	Unprocessed Red Meat
Vegetarian Burger	218 g	F	Fast Food
Vegetarian Sausage	45 g	F	Vegetarian Alternative
White Chocolate	17 g	F	Sweets
Whole Milk	250 ml	F	Milk Products
Wholegrain Bread Roll	60 g	F	Bread
Yogurt Chocolate	180 g	R	Milk Products
Yogurt Chocolate Light	180 g	R	Milk Products
Yogurt Plain	180 g	R	Milk Products
Yogurt Plain Light	180 g	R	Milk Products
Yogurt Strawberry	180 g	R	Milk Products
Yogurt Strawberry Light	180 g	R	Milk Products
